# Effect of carglumic acid with or without ammonia scavengers on hyperammonaemia in acute decompensation episodes of organic acidurias

**DOI:** 10.1186/s13023-018-0840-4

**Published:** 2018-06-20

**Authors:** Anupam Chakrapani, Vassili Valayannopoulos, Nuria García Segarra, Mireia Del Toro, Maria Alice Donati, Angeles García-Cazorla, María Julieta González, Celine Plisson, Vincenzo Giordano

**Affiliations:** 1 Metabolic Medicine Department, Great Ormond Street Hospital NHS Foundation Trust, Great Ormond Street, London, WC1N 3JH UK; 2 Hôpital Necker-Enfants Malades, Paris, France; 3 Reference Centre for Inherited Metabolic Diseases, Hôpital Robert Debré, Paris, France; 4 Servicio de Neurologíia Infantil, Hospital Vall d’Hebrón, Barcelona, Spain; 5 Reference Centre for Inherited Metabolic and Muscular Disease, Azienda Ospedaliero Universitaria Meyer, Florence, Italy; 6 Hospital Sant Joan de Déu and CIBERER, Instituto de Salud Carlos III, Barcelona, Spain; 7 Medical Affairs, Orphan Europe, Paris, France; 8 Orphan Europe, Paris, France; 9 Present Address: Sanofi Genzyme Corporation, Cambridge, MA USA; 10 Present Address: Centre Pédiatrique de Meyrin, Meyrin, Switzerland

**Keywords:** Carglumic acid, Ammonia scavengers, Organic aciduria, Hyperammonaemia, Decompensation, Extracorporeal detoxification

## Abstract

**Background:**

Hyperammonaemia is a key sign of decompensation in organic acidurias (OAs) and can contribute to severe neurological complications, thus requiring rapid treatment.

**Methods:**

A *post-hoc* analysis of two retrospective studies analysed the efficacy of carglumic acid ± ammonia (NH_3_) scavengers compared with scavengers alone for reducing plasma NH_3_ levels in patients with OAs and hyperammonaemia (plasma NH_3_ > 60 μmol/L) during decompensation episodes. NH_3_ was analysed in 12-h periods at 0–48 h and 24-h periods at 48–120 h. Treatment-emergent adverse events (TEAEs) were recorded.

**Results:**

Of 98 episodes, 38 were treated with carglumic acid (34 patients), 33 with NH_3_ scavengers (22 patients) and 27 with carglumic acid combined with NH_3_ scavengers (27 patients). Overall, 45% (carglumic acid group), 46% (NH_3_ scavengers group) and 74% (combination group) of episodes occurred in neonates. Median episode duration was 6 days for the carglumic acid and combination groups, and 9 days for the NH_3_ scavenger group. Median baseline NH_3_ level was: 199 μmol/L, carglumic acid; 122 μmol/L, NH_3_ scavengers; and 271 μmol/L, combination; 13, 30 and 11% of episodes required extracorporeal detoxification (ED), respectively. Data were censored at ED initiation. While baseline NH_3_ levels were higher in the combination and carglumic acid groups, mean reduction in NH_3_ levels to 72 h in both groups was greater than the NH_3_ scavengers’ group; reductions were greatest in the combination group.

Mean change in plasma NH_3_ vs baseline in the carglumic acid, NH_3_ scavengers and combination groups, respectively, was − 13, + 12% and − 27% at 0–12 h (*p* < 0.05 NH_3_ scavengers vs combination); − 47, − 22% and − 52% at 12–24 h (not significant); − 44, − 5% and − 61% at 24–48 h; and − 66, − 16% and − 76% at 48–72 h (*p* < 0.05 carglumic acid/combination groups vs NH_3_ scavengers for both timepoints). The number of TEAEs was similar between groups and mainly related to the disease/condition.

**Conclusions:**

Carglumic acid is a well-tolerated and efficacious treatment for OA decompensation episodes. When given alone or combined with NH_3_ scavengers, the reduction in NH_3_ was greater than with NH_3_ scavengers alone in the first 72 h.

## Background

Organic acidurias (OAs) are rare, inherited metabolic disorders, in which impaired metabolism of organic acids results in the build-up of toxic metabolites in the blood, urine and tissues [1, 2]. The classical OAs include three types of inherited disorders of branched-chain amino acids: isovaleric aciduria (IVA), methylmalonic aciduria (MMA) and propionic aciduria (PA) [1] . IVA is caused by mutations in the gene encoding isovaleryl coenzyme A (CoA) dehydrogenase, resulting in defective breakdown of leucine. MMA occurs due to a deficiency of methylmalonyl CoA mutase or due to defects of vitamin B12 metabolism. PA occurs as a result of propionyl CoA carboxylase deficiency. These disorders affect the metabolism of isoleucine, valine, methionine and threonine [1]. Secondary inhibition of the enzyme N-acetylglutamate synthase (NAGS) through accumulation of isovaleryl CoA, methylmalonyl CoA and propionyl CoA in OAs is thought to be one of the pathogenic mechanisms impeding elimination of ammonia (NH_3_) through the urea cycle, resulting in hyperammonaemia [2, 3]. In addition, the inability to maintain adequate levels of glutamine precursors secondary to a dysfunctional Krebs’ (tricarboxylic acid) cycle due to lack of succinyl CoA synthesis, impaired in both MMA and PA, is also proposed as a mechanism of hyperammonaemia in the OAs [4].

OAs typically manifest in the neonatal period, when they are characterized by toxic encephalopathy presenting within the first few days of life, with symptoms including vomiting, poor feeding and sepsis-like symptoms [5]. If untreated, the condition may progress to lethargy, seizures, coma and multiorgan failure [1]. The most common misdiagnosis of MMA and PA is sepsis. Metabolic acidosis, elevation of lactate and anion gap, urinary ketosis and disturbances of glucose metabolism may help to differentiate MMA and PA from other disorders [6]. The disease course of OA consists of acute metabolic decompensation episodes, during which aspects such as acidosis and hyperammonaemia should be considered. Importantly, these decompensation episodes are medical emergencies and may lead to severe neurological complications if not treated rapidly [1, 7]. A longer duration of hyperammonaemia and higher NH_3_ levels are associated with poorer neurological outcomes that can lead to serious consequences [1, 5, 8, 9]. Therefore, one of the main goals of treatment during OA decompensation episodes is to reduce plasma NH_3_ levels as quickly as possible [5, 9].

Current guidelines recommend various strategies for hyperammonaemia management during OA decompensation episodes, including use of NH_3_ scavengers and carglumic acid and, in the more severe cases, extracorporeal detoxification (ED) [2]. Ammonia scavengers, such as sodium phenylbutyrate and sodium benzoate, bypass the urea cycle to increase removal of NH_3_ from the blood, by conjugation of benzoate with glycine to generate hippurate, or phenylacetate with glutamine to generate phenylacetylglutamine [5, 6]. These conjugates have a higher renal clearance than NH_3_ itself, and therefore accelerate its excretion in the urine [10].

Carglumic acid is a synthetic structural analogue of N-acetylglutamate (NAG), which promotes NH_3_ detoxification by mimicking the effects of NAG on carbamoyl-phosphate synthetase I (CPS-I) [2]. CPS-I is a key enzyme of ureagenesis that catalyses the first and rate-limiting step of the urea cycle [10, 11]. A recent large, retrospective, observational study found that carglumic acid was an efficacious and well-tolerated treatment for hyperammonaemia during OA decompensation episodes [2] . The objective of the current analysis was to further evaluate the specific efficacy of the therapy in reducing raised NH_3_ levels associated with metabolic decompensation episodes in patients with OAs, without the confounding influences of NH_3_ scavengers or ED.

## Methods

### Study design and patient population

This was a *post-hoc* pooled analysis of two retrospective, observational studies. The main results from one of the studies have been published previously [2]. Data were collected from January 1995 to October 2009 in six European countries (Italy, France, Germany, The Netherlands, Spain and the United Kingdom) and Turkey.

Patients were included if they had a confirmed diagnosis of OA and hyperammonaemia (plasma NH_3_ > 60 μmol/L before treatment), treated for at least one full decompensation episode. Patients with severe hepatic insufficiency at the time of carglumic acid treatment, inherited hepatic malformation or conditions (other than OA) that might have contributed to hyperammonaemia were excluded. Mean patient age at baseline in the carglumic acid, NH_3_ scavenger and combination groups were 34.3 months, 24.6 months, and 19.9 months, respectively.

The study protocols and amendments were approved by the local independent ethics committees (IECs) and/or institutional review boards. Written informed consent/assent was obtained before data were collected. Cases in which it was not possible to obtain consent (due to death, loss to follow-up) were handled on a case-by-case basis with the relevant IEC. The studies were conducted in accordance with the principles of the Declaration of Helsinki.

### Treatments

Patients were divided into three study groups for analysis based on the treatment that they received: carglumic acid (Carbaglu®, Orphan Europe, Paris, France) alone; NH_3_ scavengers (sodium benzoate and/or sodium phenylbutyrate) alone; and carglumic acid combined with NH_3_ scavengers (combination). Due to the non-interventional, retrospective nature of the studies, oral dosing regimens of carglumic acid were not predefined, and were at the physician’s discretion. The recommended initial dose of carglumic acid in Europe (for NAGS deficiency) is 100–250 mg/kg/day [12]. In this study, the median (Q1, Q3) dose of carglumic acid in the first 24 h of treatment and the median average daily dose was 101.0 mg/kg (62.5, 200.0) and 97.9 mg/kg (66.7, 157.9), respectively, in the carglumic acid alone group, and 177.1 mg/kg (89.3, 256.4) and 98.9 mg/kg (82.6, 164.5), respectively, in the combination group. Treatment with NH_3_ scavengers was intravenously given in 72% of episodes, with use of sodium benzoate (66.7% of episodes), sodium phenylbutyrate (7.4% of episodes), or their combination (25.9% of episodes). The median dose of sodium benzoate was 257.8 (149–790) mg/kg and the median (range) dose of sodium phenylbutyrate was 282.0 (169–5625) mg/kg. In the combination group, the median doses of the NH_3_ scavengers were similar to the median doses in the NH_3_ group.

Medications of interest initiated to treat the episode were recorded. The most common treatment in all treatment groups was carnitine (90 out of 98 episodes, 91.8%). Other common treatments included arginine (11.2% of episodes), cobalamin (33.7% of episodes), glucose (25.5% of episodes), biotin (28.6% of episodes), thiamine (8.2% of episodes), and riboflavin (4.1% episodes).

### Outcomes

Data on decompensation episode characteristics, plasma NH_3_ levels and clinical symptoms were collected. The primary outcome was the reduction in plasma NH_3_ level from 0 to 120 h. Other outcomes included time to success (time to first of two consecutive measurements of plasma NH_3_ ≤ 60 μmol/L without initiation of ED, death or study withdrawal), time to 50% reduction in NH_3_ from baseline (most recent measurement prior to treatment initiation) and the shift in clinical symptoms from baseline to endpoint (last available measurement ≤18 h after the last treatment intake, or Day 15, whichever was earlier). Treatment-emergent adverse events (TEAEs) were recorded.

### Statistical analysis

Statistical analyses were performed, with continuous variables being summarized by descriptive statistics and categorical data presented by absolute and relative frequencies. Efficacy evaluations were conducted on the full analysis set, which included all decompensation episodes from patients who received at least one dose of study treatment and had a confirmed diagnosis of IVA, MMA or PA. Safety analyses were undertaken on the safety set, which included all decompensation episodes from patients who received at least one dose of study treatment.

Plasma NH_3_ was analysed in 12-h periods from 0 to 48 h and in 24-h periods from 48 to 120 h. The maximum NH_3_ value was selected for each time period. The evaluation window was ≤15 days from the first administration of treatment. NH_3_ data were censored at ED (haemodialysis/haemofiltration/peritoneal dialysis) initiation.

Statistical analyses were conducted using the Statistical Analysis Systems (SAS®) software versions 9.2/9.3 (SAS Institute, Cary, Northern Carolina, USA) and Adclin® software version TPF 3.2.2 (Adclin S.A., Paris, France).

## Results

### Episodes and treatments

**Fig. 1:**
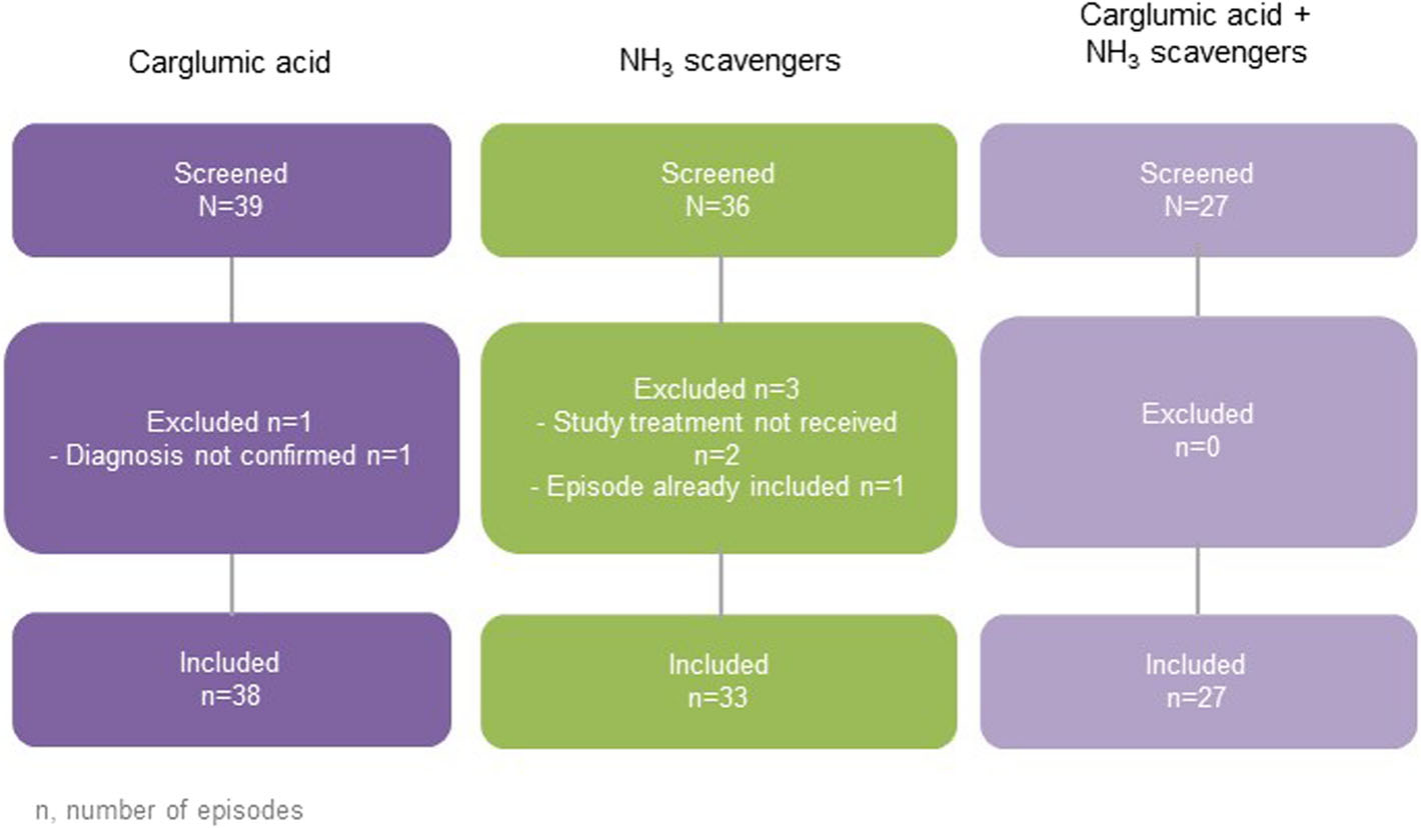
Episode disposition *N*/*n* represents the number of episodes. NH_3_: ammonia

In total, 98 episodes in 83 patients were included in the full analysis set: 38 episodes were treated with carglumic acid (*n* = 34 patients), 33 with NH_3_ scavengers (*n* = 22 patients) and 27 with a combination of both (*n* = 27 patients). The episode disposition is shown in Fig. 1. The baseline episode characteristics are displayed in Table 1. The majority of episodes (61.2%) in all treatment groups occurred in patients with a diagnosis of PA, and the proportion of episodes was higher in the NH_3_ scavengers’ group. More than 80% of episodes lasted fewer than 15 days, with a median episode duration of 6.0 days in the carglumic acid alone and combination groups, and 8.5 days in the NH_3_ scavengers’ group. Median age at start of the episode was lower, and baseline NH_3_ level was higher in the combination group than in the carglumic acid alone group or the NH_3_ scavengers’ group (Table 1). The majority (81.6%) of episodes did not require ED.

**Table 1:** Episode characteristics at baseline

	Carglumic acid	NH_3_ scavengers	Carglumic acid + NH_3_ scavengers
Episodes, *n*	38	33	27
Episodes occurring in males, *n* (%)	22 (57.9)	16 (48.5)	12 (44.4)
Episodes occurring in neonates, *n* (%)	17 (44.7)	15 (45.5)	20 (74.1)
Episodes with OA diagnosis, *n* (%)			
PA	19 (50.0)	27 (81.8)	14 (51.9)
MMA	15 (39.5)	5 (15.2)	12 (44.4)
IVA	4 (10.5)	1 (3.0)	1 (3.7)
Median (Q1, Q3) age at diagnosis, days	13.0 (5.0, 33.0)	9.5 (5.0, 19.5)	8.0 (5.0, 15.0)
Median (Q1, Q3) NH_3_ levels, μmol/L	199.0 (125.0, 295.0)	122.0 (91.0, 191.0)	270.9 (160.0, 429.0)
Median (Q1, Q3) age at start of episode, days	50.5 (4.0, 1190.0)	68.0 (4.0, 1477.0)	5.0 (4.0, 61.0)
Median (Q1, Q3) duration of ended episode, days	6.0 (3.5, 8.0)	8.5 (5.0, 12.0)	6.0 (4.0, 7.0)
Episodes requiring extracorporeal detoxification,^a^ *n* (%)	5 (13.2)	10 (30.3)	3 (11.1)

*IVA* Isovaleric aciduria, *MMA* Methylmalonic aciduria, *NH*_*3*_ Ammonia, *OA* Organic aciduria, *PA* Propionic aciduria

^a^Haemodialysis, haemofiltration and peritoneal dialysis. Full analysis set

The median duration of treatment was 4.0 days for both the carglumic acid alone and the NH_3_ scavengers’ groups, and 5.0 days for the combination group. Sodium benzoate was the most commonly used concomitant NH_3_ scavenger (alone in 66.7% of episodes and in combination with sodium phenylbutyrate in 25.9% of episodes); the median (range) total dose was 257.8 mg/kg (149–790). Sodium phenylbutyrate was used alone in 7.4% of episodes; the median (range) total dose was 282.0 mg/kg (168.6–562.5).

### Change in plasma NH_3_

**Fig. 2:**
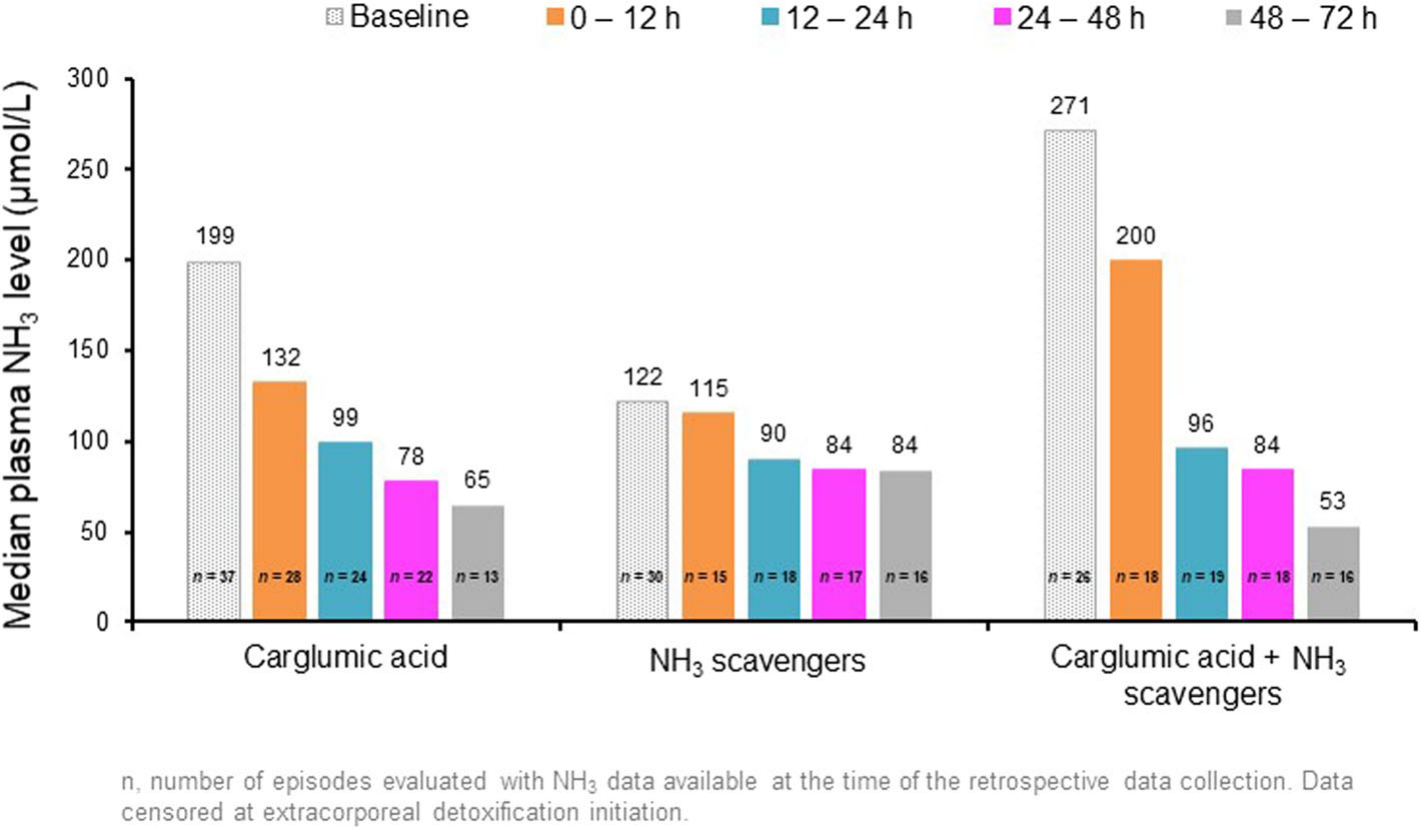
Median plasma NH_3_ levels during the first 72 h of treatment. Data were censored at extracorporeal detoxification initiation. *n* represents the number of episodes evaluated at the time of the retrospective data collection. h: hour; NH_3_: ammonia

**Fig. 3:**
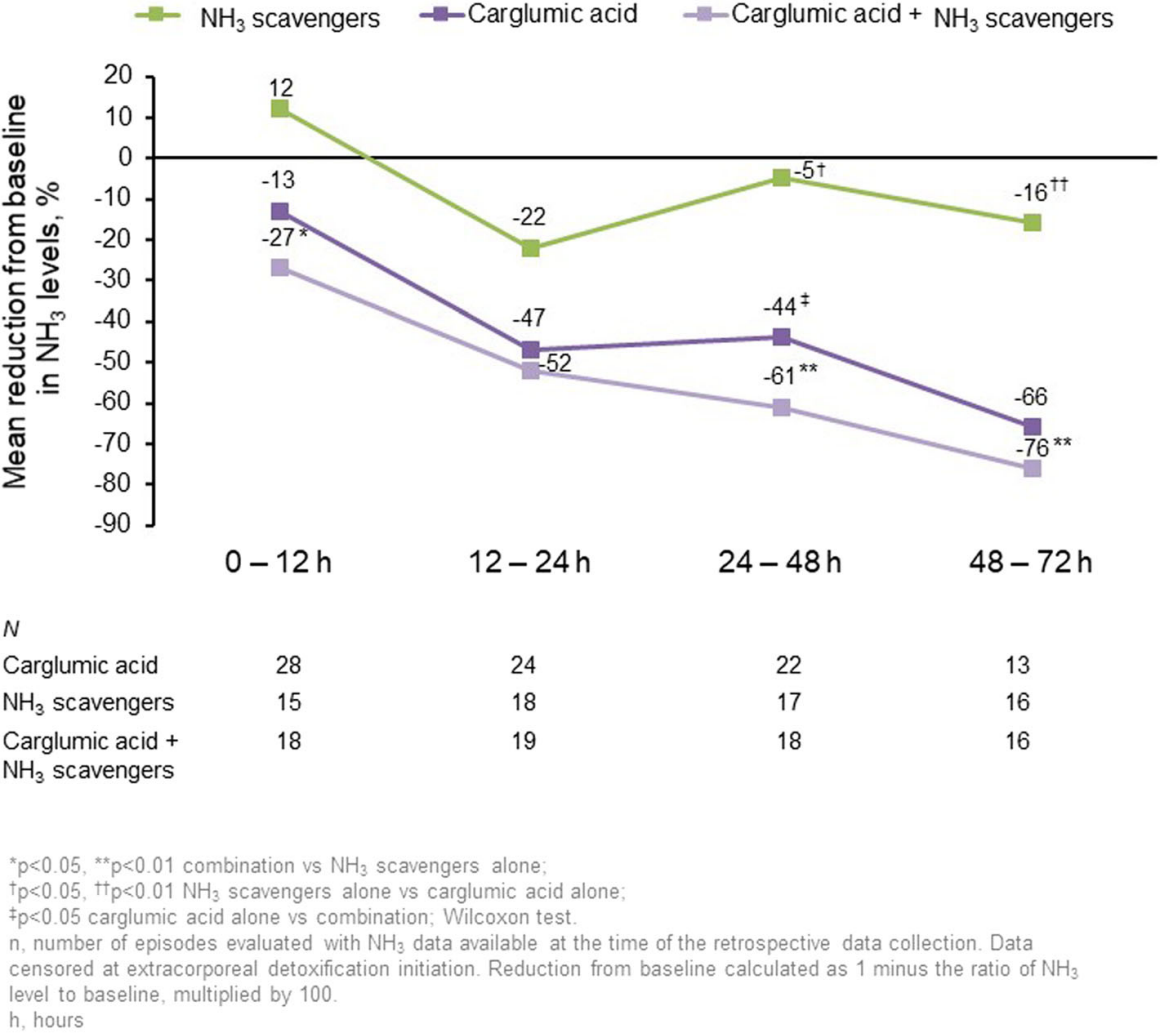
Reduction from baseline in NH_3_ levels over the first 72 h of treatment *p < 0.05 combination vs NH_3_ scavengers alone; **p < 0.01 combination vs NH_3_ scavengers alone; ^†^*p* < 0.05 NH_3_ scavengers alone vs carglumic acid alone; ^††^*p* < 0.01 NH_3_ scavengers alone vs combination; ^‡^*p* < 0.05 carglumic acid alone vs combination; Wilcoxon test. Data were censored at extracorporeal detoxification initiation. Reduction from baseline calculated as 1 minus the ratio of NH_3_ level to baseline, multiplied by 100. *N* represents the number of episodes evaluated at the time of the retrospective data collection. h: hour; NH_3_: ammonia

Daily plasma NH_3_ levels are presented in Fig. 2 and the reductions in levels from baseline during the first 72 h of treatment are displayed in Fig. 3. Up to 72 h, the mean reduction in NH_3_ from baseline in the carglumic acid alone and combination groups was greater than in the NH_3_ scavengers’ group, where both these groups had higher baseline NH_3_ levels. The reductions were greatest in the combination group, in which significantly greater reductions in plasma NH_3_ were observed versus NH_3_ scavengers alone at all timepoints, except 12–24 h. Reductions in baseline plasma NH_3_ were similar for both age categories in the carglumic acid group, and a greater reduction in plasma NH_3_ was observed for neonates in the combination group when compared with non-neonates. The reductions in plasma NH_3_ over 72 h of treatment with carglumic acid alone or in combination with NH_3_ scavengers were similar for patients with IVA, MMA and PA.

**Fig. 4:**
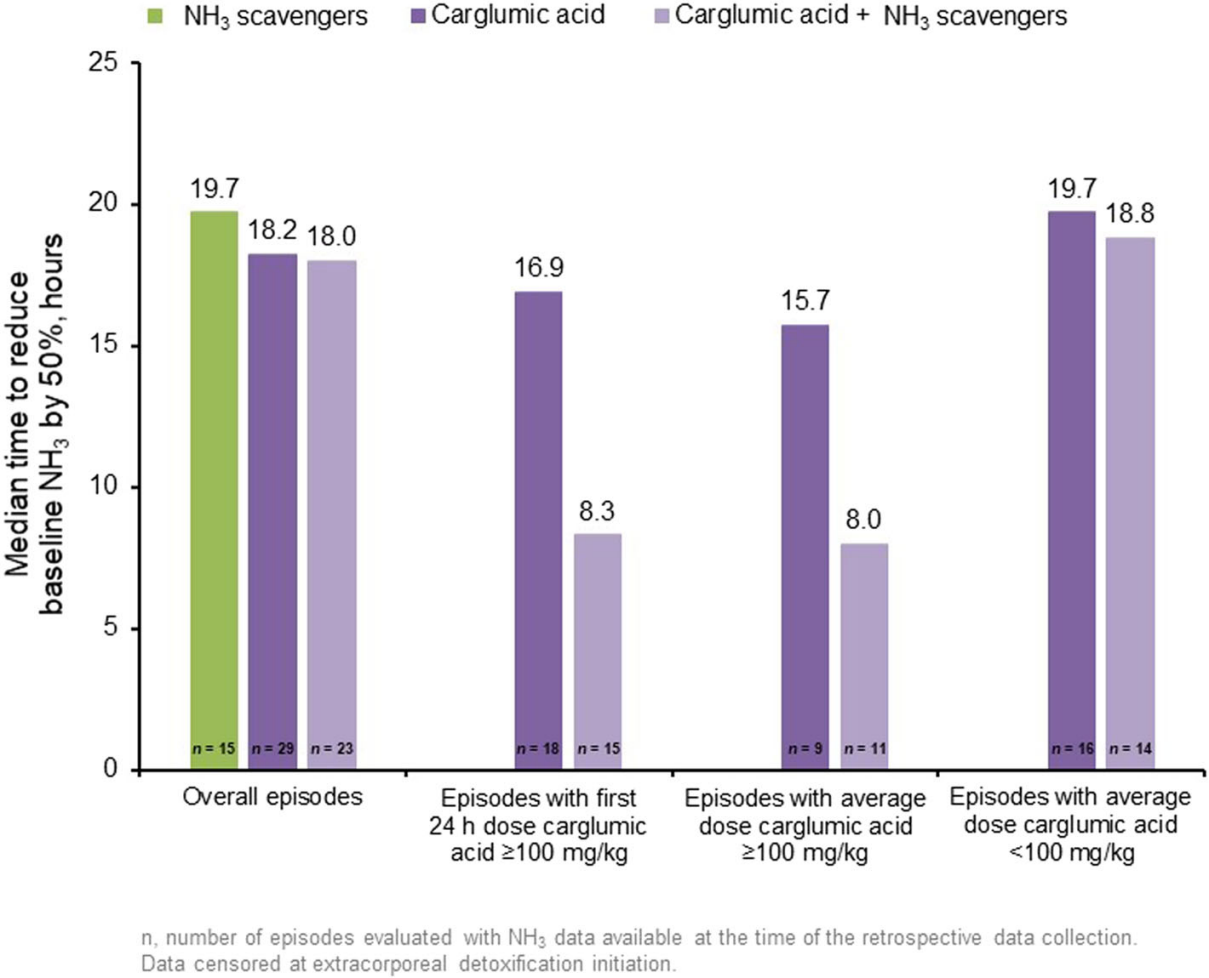
Time to reduce baseline NH_3_ levels by 50% according to the carglumic acid dose**.** Data were censored at extracorporeal detoxification initiation. Numbers represent the number of episodes evaluated at the time of the retrospective data collection. h: hour; NH_3_: ammonia

Time to success in 25% of episodes was 34 h for the carglumic acid group, 37 h for the NH_3_ scavengers’ group and 29 h for the combination group. The median time to reduce baseline NH_3_ levels by 50% is shown in Fig. 4. In the combination group, the time to halve baseline NH_3_ levels was shorter when the average dose of carglumic acid was ≥100 mg/kg than when the average dose was < 100 mg/kg in the first 24 h.

### Change in clinical symptoms

A summary of the neurological findings and feeding difficulties observed in > 10% of episodes is presented in Table 2. For the majority of symptom categories, there was a marked reduction in symptoms from baseline to study endpoint. At baseline, almost all episodes were associated with neurological findings: carglumic acid alone, 86%; NH_3_ scavengers alone, 92%; combination, 100%. By study endpoint, normal neurological status was reported for 50% (9/18) of episodes in the carglumic acid group, 36% (4/11) of episodes in the NH_3_ scavengers’ group and 45% (5/11) of episodes in the combination group. One patient in the carglumic acid alone group and one patient in the NH_3_ scavengers’ group had no neurological symptoms at baseline but developed symptoms by the study endpoint.

**Table 2:** Summary of neurological findings and feeding difficulties observed in > 10% of episodes in any treatment group at baseline

	Symptoms	Carglumic acid episodes (*n* = 38)		NH_3_ scavengers episodes (*n* = 33)		Carglumic acid + NH_3_ scavengers episodes (*n* = 27)	
		Baseline *n* episodes (%)	Endpoint *n* episodes (%)	Baseline *n* episodes (%)	Endpoint *n* episodes (%)	Baseline *n* episodes (%)	Endpoint *n* episodes (%)
Neurological findings	Abnormal movements	10 (26.3)	1 (2.6)	3 (9.1)	1 (3.0)	5 (18.5)	1 (3.7)
	Coma	6 (15.8)	1 (2.6)	1 (3.0)	0	3 (11.1)	2 (7.4)
	Lethargy	16 (42.1)	4 (10.5)	11 (33.3)	2 (6.1)	17 (63.0)	2 (7.4)
	Muscle hypotonia	15 (39.5)	7 (18.4)	12 (36.4)	1 (3.0)	19 (70.4)	4 (14.8)
	Neurological development impairment	2 (5.3)	1 (2.6)	4 (12.1)	3 (9.1)	0	0
	Seizures	2 (5.3)	0	4 (12.1)	1 (3.0)	3 (11.1)	0
	Somnolence/ asthenia	18 (47.4)	3 (7.9)	8 (24.2)	1 (3.0)	21 (77.8)	2 (7.4)
	Visual impairment	4 (10.5)	3 (7.9)	1 (3.0)	1 (3.0)	1 (3.7)	2 (7.4)^a^
Feeding difficulties	Poor feeding	15 (39.5)	4 (10.5)	13 (39.4)	0	18 (66.7)	1 (3.7)
	Vomiting	19 (50.0)	7 (18.4)	19 (57.6)	3 (9.1)	10 (37.0)	1 (3.7)

Full analysis set. *N* (%) based on the number of episodes

^a^For one patient, neurological status at baseline was erroneously marked as normal despite the patient having an ongoing history of blindness recorded at screening. The patient’s neurological status at endpoint correctly identified visual impairment. NH_3_ data was censored at extracorporeal detoxification initiation

*NH*_*3*_ ammonia

The initial manifestation of episodes was most commonly feeding difficulties, with 90% of cases in the carglumic acid group, 86% in the NH_3_ scavengers’ group and 100% of cases in the combination group presenting with such symptoms. As was seen with neurological findings, a notable shift towards normal feeding was observed with treatment, in 58% (11/19), 75% (9/12) and 80% (8/10) of episodes in the carglumic acid, NH_3_ scavengers and combination groups, respectively.

### Safety

Overall, there were fewer adverse events (AEs) in the combination group (*n* = 21) than in the carglumic acid group (*n* = 61) or the NH_3_ scavengers’ group (*n* = 97). One-hundred and fifty seven TEAEs were reported, including 51 serious TEAEs (Table 3) – 54 and 13 in the carglumic acid group, 85 and 31 in the NH_3_ scavengers’ group, and 18 and 7 in the combination group, respectively. Overall, the AEs reported were largely related to the disease/condition (i.e. metabolic decompensation) rather than drug toxicity. A total of 22 individual fatal TEAEs that could each have resulted in death occurred in 10 patients/episodes. Three fatal AEs occurred in three patients in the carglumic acid group; 13 fatal AEs occurred in four patients in the NH_3_ scavengers’ group; and six fatal AEs occurred in three patients in the combination group.

**Table 3:** Treatment-emergent adverse events

	Carglumic acid (*n* = 38)	NH_3_ scavengers (*n* = 33)	Carglumic acid + NH_3_ scavengers (*n* = 27)
Number of TEAEs	54	85	18
Number of drug-related^a^ TEAEs	18	1	6
Number of serious TEAEs	13	31	7
Number of drug-related^b^ serious TEAEs	5	0	1
TEAEs leading to death	3	13	6
TEAEs occurring in ≥10% of episodes in any treatment group, *n* episodes (%)			
Coagulopathy	1 (2.6)^b^	5 (15.2)	0
Thrombocytopenia	2 (5.3)^b^	7 (21.2)	1 (3.7)
Hyperglycaemia	1 (2.6)	5 (15.2)	1 (3.7)
Hypocalcaemia	1 (2.6)^b^	4 (12.1)	0
Hypokalaemia	1 (2.6)	6 (18.2)	0
Respiratory distress	0	4 (12.1)	0

*NH*_*3*_ Ammonia, *TEAE* Treatment-emergent adverse event

^a^Drug-relatedness in the combination group refers exclusively to carglumic acid treatment and not NH_3_ scavenger treatment

^b^Considered to be drug-related in one episode/patient. *n* represents the number of episodes evaluated

## Discussion

Hyperammonaemia is one of the most severe, life-threatening symptoms in OA metabolic decompensation episodes [6]. Acute hyperammonaemia is a medical emergency and early, rapid reduction of plasma NH_3_ levels, using both pharmacological treatments and non-pharmacological methods available, is required to limit the potential unfavourable neurological symptoms encountered in patients with OA, as well as to reduce the risk of fatal outcomes [5, 6, 13]. Treatment with NH_3_ scavengers may be beneficial by increasing the reduction of plasma NH_3_ levels; however, caution is advised when using in MMA and PA due to potential NH_3_ toxicity by blocking the urea cycle through sequestration of CoA [6, 14, 15]. Additionally, sodium phenylbutyrate acts through conjugation of glutamine, and may worsen the glutamine depletion that is one of the proposed mechanisms of hyperammonaemia and energy depletion in the OAs [6]. Therefore, sodium phenylbutyrate is not preferred in the treatment of hyperammonaemia in the OAs [6, 16]. Furthermore, treatment with these agents at high doses may increase serum sodium and decrease serum potassium levels [15, 17]. In particularly severe hyperammonaemia, or when treatment with other methods has failed, ED by haemodialysis, haemofiltration or peritoneal dialysis may also be required [5, 6]. Caution is recommended when using ED, as the procedure is invasive, has a risk of technical failure, and can cause infectious and haemodynamic complications in infants, and use of ED is limited by local facilities [5, 6].

The efficacy and safety of carglumic acid during decompensation episodes in OAs has been explored in our previous publication [2]. This analysis aimed to evaluate the efficacy of carglumic acid and its tolerability when used with or without NH_3_ scavengers, for the treatment of hyperammonaemia during OA decompensation episodes. The data in this analysis demonstrate that carglumic acid is efficacious in reducing NH_3_ levels, and produces greater reductions in plasma NH_3_ levels than NH_3_ scavengers alone during the first 72 h of metabolic decompensation episodes. Reductions in plasma NH_3_ following treatment were accompanied by improvements in clinical symptoms and neurological status. More significant reductions in NH_3_ were observed in the combination group. This could be attributed to the higher dosage of carglumic acid administered within the first 24 h or may suggest a potentially greater clinical impact of combination therapy compared with either NH_3_ scavengers or carglumic acid alone. Reductions in ammonia levels may appear to be more pronounced in the groups where patients had higher baseline levels. It is worth noting that differences in local practice may also reflect the higher percentage of episodes treated via ED within the NH_3_ scavengers’ group compared with the carglumic acid or combination groups.

The higher baseline plasma NH_3_ levels reported for the combination group were also closer to the range associated with irreversible neurological deficits following prolonged exposure and, therefore, these reductions might be more clinically relevant [8].

Conversely, the baseline plasma NH_3_ levels in the carglumic acid and NH_3_ scavengers’ group were less elevated, and data from this study do not demonstrate if the relative reduction reported will translate into a clinically meaningful improvement in a patient with a higher baseline plasma NH_3_ level. Analysis of these findings for patients with IVA was also limited by the low number of IVA patients included in the study; further investigation is required for the use of carglumic acid and NH_3_ scavengers in this population.

This exploratory analysis suggests that the reduction of NH_3_ levels may be more rapid with carglumic acid treatment than with NH_3_ scavengers; this is particularly important during the first hours of an episode in order to limit potential complications. The time to reduce baseline NH_3_ by 50% was shortest in patients who received a first 24-h dose or average daily dose of carglumic acid ≥100 mg/kg, which is in line with the recommended dose for the treatment (100–250 mg/kg) [18]. More episodes required treatment with ED in the NH_3_ scavengers alone group than in the carglumic acid alone or combination groups (30.3, 13.2 and 11.1%, respectively). It is possible that the more rapid reductions in plasma NH_3_ observed in the carglumic acid groups may have led to a decision not to initiate ED.

Overall, treatment with carglumic acid was well tolerated. There were fewer TEAEs in the combination group than in the carglumic acid and NH_3_ scavengers monotherapy groups, and the reported TEAEs were mostly related to the condition rather than drug toxicity. The categories of drug relatedness were different between the two studies analysed, and relatedness in the combination group referred exclusively to carglumic acid and not NH_3_ scavengers. These results suggest that, in addition to improved efficacy in the reduction of plasma NH_3_ levels, use of a combination of carglumic acid and NH_3_ scavengers may also reduce the risk of AEs in some patients.

This was a retrospective, exploratory analysis, with missing data for a number of patients for some timepoints, and without control groups. We acknowledge these shortcomings and that they would best be addressed in a study with a controlled and prospective design.

However, the study provides real-world data on the use of carglumic acid with or without NH_3_ scavengers for the treatment of metabolic decompensation episodes. Data from this analysis are consistent with previous published data supporting the use of carglumic acid in acute hyperammonaemia to reduce plasma NH_3_ and the need for peritoneal dialysis and haemodialysis [10].

## Conclusions

The results of this analysis indicate that carglumic acid, either alone or combined with NH_3_ scavengers, produces greater reductions in plasma NH_3_ levels than NH_3_ scavengers alone in episodes of hyperammonaemia occurring in metabolic decompensation in patients with OAs. These analyses support the early use of carglumic acid in the treatment of acute hyperammonaemia in paediatric patients with OA.

## Abbreviations

AE: Adverse event
CoA: Coenzyme A
CPS-I: Carbamoyl-phosphate synthetase I
ED: Extracorporeal detoxification
IEC: Independent ethics committee
IVA: Isovaleric aciduria
MMA: Methylmalonic aciduria
NAG: N-acetylglutamate
NAGS: N-acetylglutamate synthase
NH_3_: Ammonia
OA: Organic aciduria
PA: Propionic aciduria
TEAE: Treatment-emergent adverse event
